# Thiamine as an adjunctive therapy in cardiac surgery: a randomized, double-blind, placebo-controlled, phase II trial

**DOI:** 10.1186/s13054-016-1245-1

**Published:** 2016-03-14

**Authors:** Lars W. Andersen, Mathias J. Holmberg, Katherine M. Berg, Maureen Chase, Michael N. Cocchi, Christopher Sulmonte, Julia Balkema, Mary MacDonald, Sophia Montissol, Venkatachalam Senthilnathan, David Liu, Kamal Khabbaz, Adam Lerner, Victor Novack, Xiaowen Liu, Michael W. Donnino

**Affiliations:** Center for Resuscitation Science, Department of Emergency Medicine, Beth Israel Deaconess Medical Center, 330 Brookline Avenue, Boston, MA 02215 USA; Department of Anesthesiology, Aarhus University Hospital, Nørrebrogade 44, 8000 Aarhus C, Denmark; Research Center for Emergency Medicine, Aarhus University Hospital, Nørrebrogade 44, 8000 Aarhus C, Denmark; Department of Medicine, Division of Pulmonary and Critical Care, Beth Israel Deaconess Medical Center, 330 Brookline Avenue, Boston, MA 02215 USA; Department of Anesthesia Critical Care, Division of Critical Care, Beth Israel Deaconess Medical Center, 330 Brookline Avenue, Boston, MA 02215 USA; Department of Surgery, Division of Cardiothoracic Surgery, Beth Israel Deaconess Medical Center, 330 Brookline Avenue, Boston, MA 02215 USA; Department of Anesthesia, Beth Israel Deaconess Medical Center, 330 Brookline Avenue, Boston, MA 02215 USA; Clinical Research Center, Soroka University Medical Center, POB 151, Beer-Sheva, 84965 Israel; Faculty of Health Sciences, Ben-Gurion University, POB 151, Beer-Sheva, 84965 Israel

**Keywords:** Thiamine, Lactate, Cardiac surgery, Coronary artery bypass grafting, Pyruvate dehydrogenase, Aerobic, Anaerobic, Metabolism, Oxygen consumption

## Abstract

**Background:**

Thiamine is a vitamin that is essential for adequate aerobic metabolism. The objective of this study was to determine if thiamine administration prior to coronary artery bypass grafting would decrease post-operative lactate levels as a measure of increased aerobic metabolism.

**Methods:**

We performed a randomized, double-blind, placebo-controlled trial of patients undergoing coronary artery bypass grafting. Patients were randomized to receive either intravenous thiamine (200 mg) or placebo both immediately before and again after the surgery. Our primary endpoint was post-operative lactate levels. Additional endpoints included pyruvate dehydrogenase activity, global and cellular oxygen consumption, post-operative complications, and hospital and intensive care unit length of stay.

**Results:**

Sixty-four patients were included. Thiamine levels were significantly higher in the thiamine group as compared to the placebo group immediately after surgery (1200 [683, 1200] nmol/L vs. 9 [8, 13] nmol/L, *p* < 0.001). There was no difference between the groups in the primary endpoint of lactate levels immediately after the surgery (2.0 [1.5, 2.6] mmol/L vs. 2.0 [1.7, 2.4], *p* = 0.75). Relative pyruvate dehydrogenase activity was lower immediately after the surgery in the thiamine group as compared to the placebo group (15 % [11, 37] vs. 28 % [15, 84], *p* = 0.02). Patients receiving thiamine had higher post-operative global oxygen consumption 1 hour after the surgery (difference: 0.37 mL/min/kg [95 % CI: 0.03, 0.71], *p* = 0.03) as well as cellular oxygen consumption. We found no differences in clinical outcomes.

**Conclusions:**

There were no differences in post-operative lactate levels or clinical outcomes between patients receiving thiamine or placebo. Post-operative oxygen consumption was significantly increased among patients receiving thiamine.

**Trial registration:**

clinicaltrials.gov NCT02322892, December 14, 2014

**Electronic supplementary material:**

The online version of this article (doi:10.1186/s13054-016-1245-1) contains supplementary material, which is available to authorized users.

## Background

Over 230,000 patients in the United States undergo coronary artery bypass grafting (CABG) each year [1]. Although mortality in this patient population is relatively low, complications increasing both morbidity and length of stay remain significant [2–4]. Lactate elevation, a marker of anaerobic metabolism, is commonly seen after major cardiac surgery and multiple studies have found an association between elevated post-operative lactate levels and increased morbidity and mortality [5–10]. Elevated lactate has also been associated with poor outcome in other forms of critical illness, such as sepsis [11, 12]. The rise in lactate in these settings is thought to be secondary to inadequate oxygen delivery and/or a deficit in aerobic metabolism [13]. The importance of this decrease in aerobic metabolism is also supported by an association between low oxygen consumption and poor outcome in critically ill states including sepsis, high-risk surgery, and post-cardiac arrest patients [14–16].

**Fig. 1:**
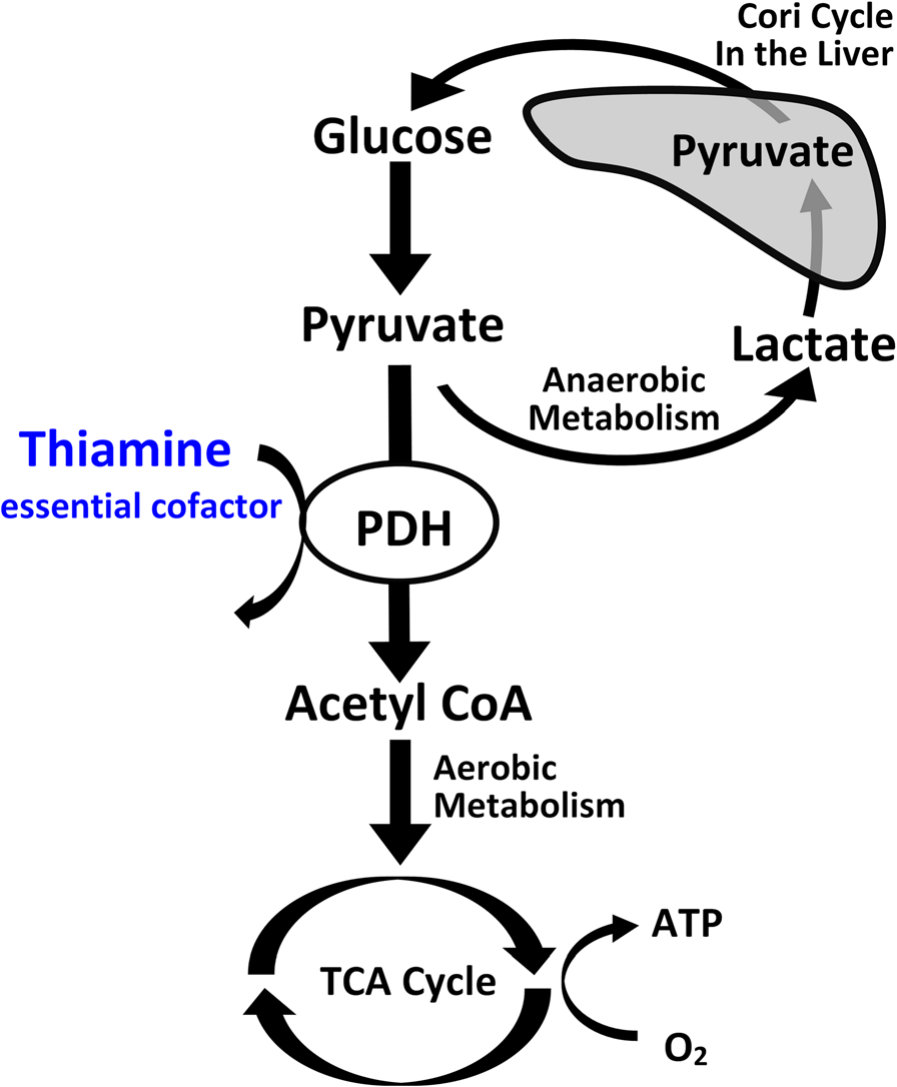
Simplified graphical presentation of PDH’s and thiamine’s role in aerobic metabolism. Aerobic metabolism occurs when pyruvate enters the mitochondria through pyruvate decarboxylation to acetyl-coenzyme A, facilitated by the rate-limiting enzyme pyruvate dehydrogenase (PDH). Adapted with permission from Andersen et al. [12]. *ATP* adenosine triphosphate, CoA coenzyme A, *TCA* tricarboxylic acid

Aerobic metabolism occurs when pyruvate enters the mitochondria through pyruvate decarboxylation to acetyl-coenzyme A. This entry into the mitochondria (and tricarboxylic acid [TCA] cycle) is facilitated by the rate-limiting enzyme pyruvate dehydrogenase (PDH) (Fig. 1) [17, 18]. Decreased PDH activity may cause a shift toward anaerobic metabolism and potentially play a role in the metabolic changes (i.e., elevated lactate) seen in patients undergoing CABG with cardiopulmonary bypass [19–24]. Thiamine (vitamin B1) is an essential co-factor for PDH function. In the absence of thiamine, the conversion of pyruvate to acetyl-coenzyme A is inhibited, cellular oxygen consumption is decreased, and lactate is produced [25]. Thiamine deficiency is common after cardiac surgery and post-operative thiamine levels have been found to be inversely associated with post-operative lactate levels [19, 26].

We hypothesized that thiamine administration prior to surgery would decrease post-operative lactate levels in patients undergoing CABG by increasing PDH activity and oxygen consumption, ultimately leading to improved clinical outcomes.

## Methods

### Design and setting

This was a single-center, randomized, double-blind, placebo-controlled, phase II trial of thiamine in patients undergoing CABG with cardiopulmonary bypass. Patients were enrolled at Beth Israel Deaconess Medical Center, Boston, MA, USA - a tertiary care center with more than 450 CABG surgeries performed annually. The study was approved by The Committee on Clinical Investigations at Beth Israel Deaconess Medical Center (protocol number: 2014-P000257) and patients provided written informed consent prior to enrollment. The trial was registered at clinicaltrials.gov (NCT02322892) and funded by the American Heart Association (15CRP22830000).

### Study population

We enrolled consecutive patients between January 2015 and July 2015. We included adult patients (≥21 years) scheduled for CABG with or without concomitant valve procedures. Due to unpublished preliminary data suggesting that patients with a higher European System for Cardiac Operative Risk Evaluation (EuroSCORE) II score have higher post-operative lactate levels, we only included patients with a EuroSCORE II score > 1.5 %. We excluded patients based on the following criteria: (1) current thiamine supplementation or known clinical indication for thiamine (e.g., alcohol dependency), (2) known allergy to thiamine, (3) emergent or salvage CABG (as defined by the Society of Thoracic Surgeons [27]), (4) CABG without cardiopulmonary bypass (“off-pump” surgery), and (5) protected population (pregnant women, prisoners, and the intellectually disabled).

### Randomization, study drug and blinding

Patients were randomized in blocks of four in a 1:1 ratio to receive thiamine or placebo. The randomization was stratified by “high” (>4 %) vs. “low” (≤4 %) EuroSCORE II to minimize baseline heterogeneity between the two groups. Patients received 200 mg thiamine in 50 mL 0.9 % saline or matching placebo (50 mL 0.9 % saline) immediately before the surgery and again immediately after the surgery upon arrival in the intensive care unit. The placebo and the thiamine were identical in appearance; patients, healthcare personnel, and the research team remained blinded throughout the study period.

### Endpoints and data collection

The primary endpoint was post-operative lactate levels collected upon arrival to the intensive care unit. Key secondary endpoints included PDH activity, post-operative complications, intensive care unit and hospital length of stay, and mortality. Additional endpoints included lactate levels 6 hours after the surgery, time on mechanical ventilation (defined as the time from the end of surgery until extubation), time on vasopressors (defined as the time from the end of surgery until discontinuation of all vasopressors [epinephrine, norepinephrine, phenylephrine, dopamine, and vasopressin] for at least 6 hours), and cellular and global oxygen consumption (see below). Post-operative complications before hospital discharge included: new atrial fibrillation (requiring treatment or lasting for > 24 hours), renal failure (requiring new dialysis), stroke as defined by Sacco et al. [28], myocardial infarction using the universal definition [29], acute respiratory distress syndrome using the Berlin definition [30], infection (requiring new antibiotics), and documented delirium.

Before the surgery, we recorded demographic data and past medical history including the New York Heart Association (NYHA) classification [31] and the Canadian Cardiovascular Society grading of angina pectoris [32]. We also calculated the EuroSCORE II [33], which is a validated pre-operative score to predict post-operative morbidity and mortality [34–36]. The score utilizes information regarding the patient (age, gender, and co-morbidities) and current cardiac status (NYHA class, presence of unstable angina, left ventricular function and more), as well as factors related to the operation (urgency and type of intervention).

All data were collected by a trained research assistant according to a detailed, pre-defined data dictionary and all outcome variables were verified by a physician. Data were entered into a secure, online database (Research Electronic Data Capture [RedCAP]) [37].

### Blood samples

Blood samples were obtained immediately before administration of the first study dose, upon arrival to the intensive care unit (immediately before administration of the second study dose), and again 6 hours later. Upon collection, blood was sent to the hospital’s clinical laboratory for lactate measurements (Rapidlab 1265, Siemens Healthcare Diagnostics Inc., Tarrytown, NY, USA). Fresh whole blood collected in ethylenediaminetetraacetic acid tubes was used for measurement of PDH and cellular oxygen consumption (see below). The remaining blood was centrifuged at 3500 rpm for 10 minutes. Plasma and serum were aliquoted into light-protected cryotubes and frozen at −80 °C for later measurements of thiamine levels. All blood samples were collected from pre-existing arterial lines except one draw that was collected from a pre-existing central venous line.

### Thiamine levels and pyruvate dehydrogenase

Thiamine levels were measured in plasma via liquid chromatography-tandem mass spectrometry by Quest Diagnostics (Nichols Institute, Chantilly, VA, USA). Absolute thiamine deficiency was determined using a previously established standard laboratory reference range from Quest Diagnostics; specifically, absolute thiamine deficiency was defined as a level ≤ 7 nmol/L. If a thiamine level was undetectable (i.e., < 7 nmol/L) a value of 7 nmol/L was imputed.

Peripheral blood mononuclear cells (PBMCs) were isolated from fresh whole blood using a density gradient separation method (Ficoll-Paque premium, GE Healthcare Bio-Science Corp., Piscataway, NJ, USA). PDH activity and quantity were then measured after disruption of the mitochondrial membrane via an immunocapture and microplate-based assay as previously described [38, 39]. PDH specific activity was calculated as PDH activity/ln (PDH quantity). PDH activity and quantity are expressed in OD/min/mg protein where OD indicates the absorbance (optical density). Post-operative PDH values are expressed as relative to the pre-operative PDH value (i.e., [PDH_post-surgery_/PDH_pre-surgery_] × 100 %).

### Global and cellular oxygen consumption

Based on equipment availability, we measured global oxygen consumption (VO_2_) in a subset of patients using a compact anesthesia monitor, which was connected to the ventilator tubing via a ventilator adapter with an attached gas sampling line (General Electric, Fairfield, CT, USA). This device measures VO_2_ continuously on a breath-by-breath basis using an incorporated pneumotachograph to measure the volume of gas being exchanged, and a paramagnetic analyzer to detect differences in inspired and expired oxygen [40, 41]. The monitor does not measure VO_2_ when the fraction of inspired oxygen is > 85 %. VO_2_ was recorded every 5 minutes from arrival in the intensive care unit until extubation or until the second post-operative blood draw. For the analysis, we used data from 1 hour after the surgery until 4 hours after the surgery. We removed non-physiological outliers (VO_2_ < 150 mL/min and VO_2_ > 700 mL/min) and very inconsistent values (i.e., single values that changed substantially within a short timeframe). VO_2_ was normalized to body weight.

Based on laboratory personnel and equipment availability (as the measurement must be performed at the time of sample collection), we measured the cellular oxygen consumption rate (OCR) in PBMCs on a subset of the enrolled patients. The complete mitochondrial respiration profile was measured using the XF Cell Mito Stress Test Kit in an XF^e^96 Extracellular Flux Analyzer (Seahorse Bioscience, North Billerica, MA, USA). This technology has been described in detail elsewhere [42]. For this manuscript, we report basal cellular respiration as well as maximal cellular respiration (see Additional file 1). The value of interest was the immediate post-surgery OCR relative to the pre-surgery OCR (i.e., [OCR_post-surgery_/OCR_pre-surgery_] × 100 %).

### Sample size calculation and statistical analysis

The sample size for the current study was based on unpublished preliminary data from a small open-label trial of thiamine administration and historical observational data (see Additional file 1). Based on this, we estimated that the placebo group would have a mean post-operative lactate level of 3.6 mmol/L and that the thiamine group would have a mean lactate level of 2.6 mmol/L with both groups having a standard deviation of 1.4 mmol/L. Based on these estimates, 32 patients in each group provide 80 % power for a two-sided *t* test at an alpha level of 0.05.

Descriptive statistics were used to characterize the study population; continuous variables are presented as means with standard deviations or medians with 1^st^ and 3^rd^ quartiles depending on the normality of the data. Categorical variables are presented as counts with relative frequencies. Continuous data were compared between the groups using a two-sample *t* test or Wilcoxon rank sum test depending on normality of the data. Categorical data were compared between groups using Fisher’s exact test. The primary analysis was a comparison of lactate levels immediately after the surgery. Secondarily, we used repeated measures analysis to analyze lactate levels immediately and 6 hours after surgery using an unstructured variance-covariance structure adjusting for the pre-surgery level as well as the stratification factor (i.e., low vs. high EuroSCORE II) [43]. PDH levels and VO_2_ were similarly analyzed using a repeated measures approach. For the latter analysis, we assumed a 1^st^ order autoregressive variance-covariance structure. OCR was compared between groups using linear regression with adjustment for the stratification factor. Right-skewed variables (lactate, PDH values and OCR) were log-transformed before analysis.

We performed three subgroup analyses for the primary endpoint; according to the stratification variable, according to the pre-surgery thiamine level (dichotomized as above/below the median) and according to whether the patient had diabetes or not as a relatively large proportion of patients with diabetes may have thiamine deficiency [44, 45].

Analyses were conducted on a modified intention-to-treat basis including only those subjects who received the first dose of the study drug [46]. Data were complete for the primary endpoint and no imputations were done for missing secondary outcomes. All statistical analyses were pre-defined (i.e., planned before unblinding of the data) unless otherwise specified. All hypothesis tests were two-sided, with a significance level of *p* < 0.05. Given the pilot nature of the current study, no adjustments were made for multiple testing and all secondary outcomes should therefore be considered exploratory. Statistical analyses were conducted with the use of SAS software, version 9.4 (SAS Institute Inc., Cary, NC, USA).

## Results

### Study population and thiamine levels

**Fig. 2:**
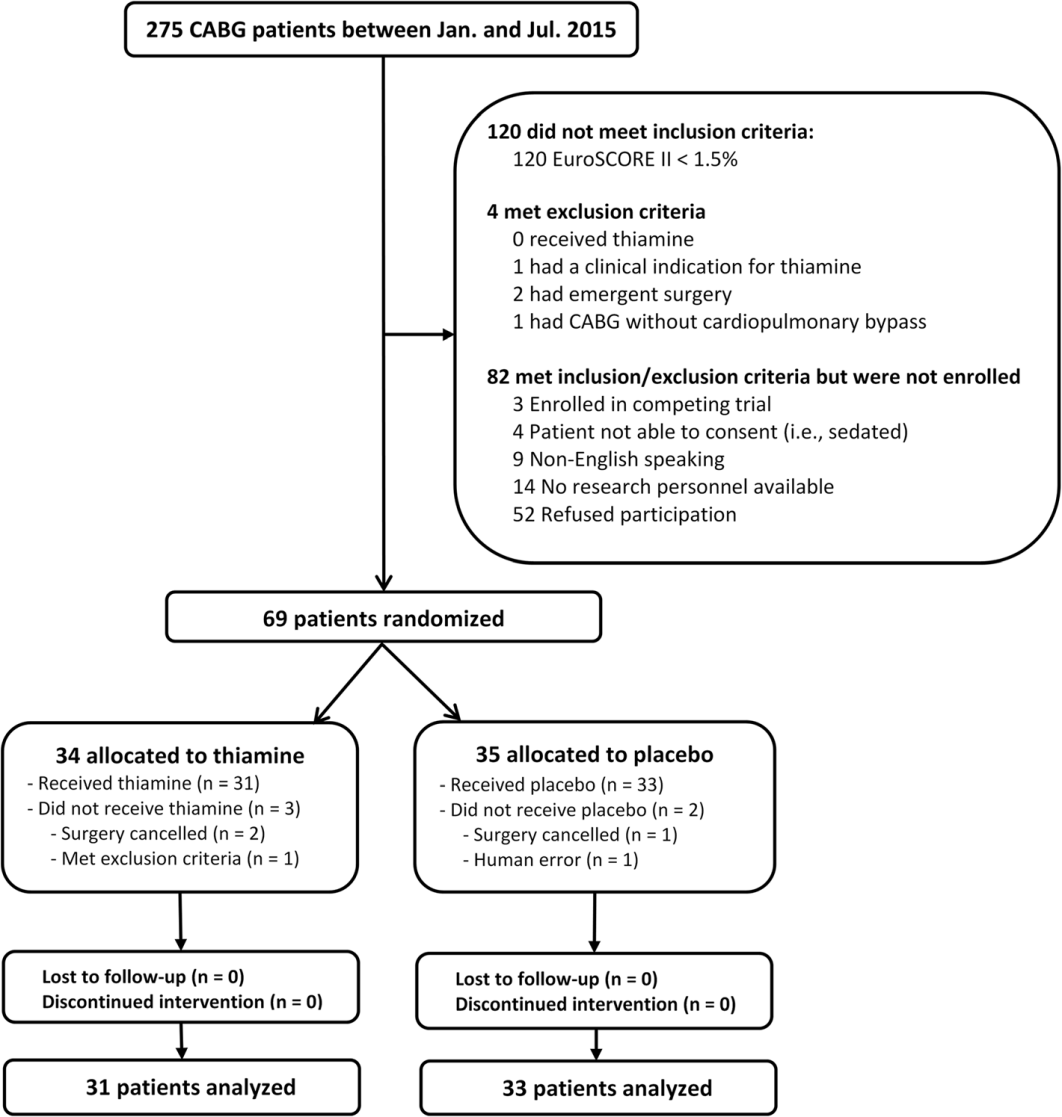
CONSORT diagram. Out of 275 patients screened, 69 were randomized and 64 were analyzed per the modified intention-to-treat analysis. No patients discontinued the intervention or were lost to follow-up. *CABG* coronary artery bypass grafting, *EuroSCORE* European System for Cardiac Operative Risk Evaluation

Of 275 patients screened, 69 were randomized and 64 received the first study dose and were therefore included in the analysis (Fig. 2). One patient was scheduled for CABG and valve surgery but only received valve surgery. Per our pre-defined analysis plan, this patient was not excluded. The baseline characteristics of the patients are provided in Table 1 and surgical characteristics in Table 2. There were no statistically significant differences in surgical characteristics between the groups. There was no difference in the pre-operative lactate levels between the groups (1.2 mmol/L [1.0, 1.3] vs. 1.1 mmol/L [1.0, 1.3]).

**Table 1:** Baseline characteristics of the study patients^a^

	Thiamine (n = 31)	Placebo (n = 33)
Demographics		
Age (years)	71 (67, 75)	73 (68, 79)
Sex (female)	6 (19)	10 (30)
Body mass index (kg/m^2^)	29 (26, 33)	28 (25, 29)
Race		
White	31 (100)	32 (97)
Black	0 (0)	1 (3)
EuroSCORE II (%)	3.4 (2.1, 5.2)	2.3 (1.9, 4.5)
EuroSCORE II > 4.0 %	9 (29)	12 (36)
Cardiac past medical history		
MI/PCI	14 (45)	12 (36)
Atrial fibrillation	7 (23)	5 (15)
Previous cardiac surgery	0 (0)	2 (6)
Chronic heart failure	4 (13)	8 (24)
Valve disease	12 (39)	8 (24)
Ejection fraction (%)	53 (40, 60)	55 (45, 60)
Current NYHA class^b^		
I	5 (16)	1 (3)
II	12 (29)	14 (42)
III	14 (45)	15 (45)
IV	0 (0)	3 (9)
Current angina class^b^		
No symptoms	13 (42)	14 (42)
I	0 (0)	1 (3)
II	3 (10)	5 (15)
III	7 (23)	7 (21)
IV	8 (26)	8 (18)
Other past medical history		
Pulmonary disease	3 (10)	5 (15)
Diabetes	14 (45)	14 (42)
Insulin dependent	7 (50)	6 (43)
Non-insulin dependent	7 (50)	8 (57)
Renal disease	7 (23)	11 (33)
Cancer	1 (3)	2 (6)
Pre-operative laboratory values		
White blood count (×10^3^)	8.2 (6.8, 8.9)	7.5 (5.7, 10.0)
Hemoglobin (g/dL)	12.9 (11.5, 14.4)	13.0 (11.2, 14.2)
Creatinine (mg/dL)	1.1 (0.8, 1.6)	1.1 (0.8, 1.3)
Glucose (mg/dL)	131 (108, 164)	114 (94, 145)
Pre-surgical characteristics		
Status		
Elective	14 (45)	12 (36)
Urgent	17 (55)	21 (65)
Location prior to surgery		
Home	13 (42)	9 (27)
Ward	15 (48)	24 (73)
Intensive care unit	3 (10)	0 (0)

*Abbreviations*: *EuroSCORE* European System for Cardiac Operative Risk Evaluation, *MI* myocardial infarction, *PCI* percutaneous coronary intervention, *NYHA* New York Heart Association

^a^Categorical variables are presented as count (frequency) and continuous variables as median (quartiles)

^b^Defined as the worst classification within the last 2 weeks

**Table 2:** Surgical characteristics of the study patients^a^

	Thiamine (n = 31)	Placebo (n = 33)	*p* value
Vessels grafted			0.14
1	5 (17)	6 (18)	
2	0 (0)	5 (15)	
3	13 (43)	14 (42)	
4	11 (37)	8 (24)	
5	1 (3)	0 (0)	
Valve surgery			0.15
None	17 (55)	22 (67)	
Aortic	13 (42)	7 (21)	
Mitral	1 (3)	4 (12)	
Other procedure	2 (6)	1 (3)	0.61
Intra-operative complication(s)	2 (6)	0 (0)	0.23
Length of surgery (min)	211 (186, 251)	210 (191, 226)	0.56
Bypass time (min)	95 (69, 118)	80 (72, 96)	0.24
Cross-clamp time (min)	77 (54, 98)	63 (54, 76)	0.13
Received red blood cells	9 (29)	5 (15)	0.23
Fluids/transfusion (mL)			
Saline	1000 (500, 1500)	1000 (400, 1800)	0.84
Lactated Ringer’s	1700 (1000, 2000)	1600 (1200, 2100)	0.85
Cell saver	400 (300, 450)	250 (300, 430)	0.49
Estimated blood loss (mL)^b^	200 (123, 500)	200 (123, 500)	0.79
Urine output (mL)	350 (265, 600)	515 (325, 710)	0.13

^a^Categorical variables are presented as count (frequency) and continuous variables as median (quartiles)

^b^As estimated by the surgeon. Missing on seven patients

Thiamine levels were similar between the thiamine and placebo group prior to the surgery (14 nmol/L [11, 18] vs. 14 nmol/L [12, 18]) with one patient in the thiamine group and zero patients in the placebo group having thiamine deficiency. Thiamine levels were substantially higher in the thiamine group as compared to the placebo group immediately after surgery (1200 nmol/L [683, 1200] vs. 9 nmol/L [8, 13], *p* < 0.001) and 6 hours after the surgery (1200 nmol/L [872, 1200] vs. 10 nmol/L [7, 15], *p* < 0.001). No patients in the thiamine group were deficient after the surgery as compared to three (9 %) in the placebo group immediately after the surgery (p = 0.24) and five (15 %) patients 6 hours after the surgery (*p* = 0.05).

### Lactate and PDH values

**Fig. 3:**
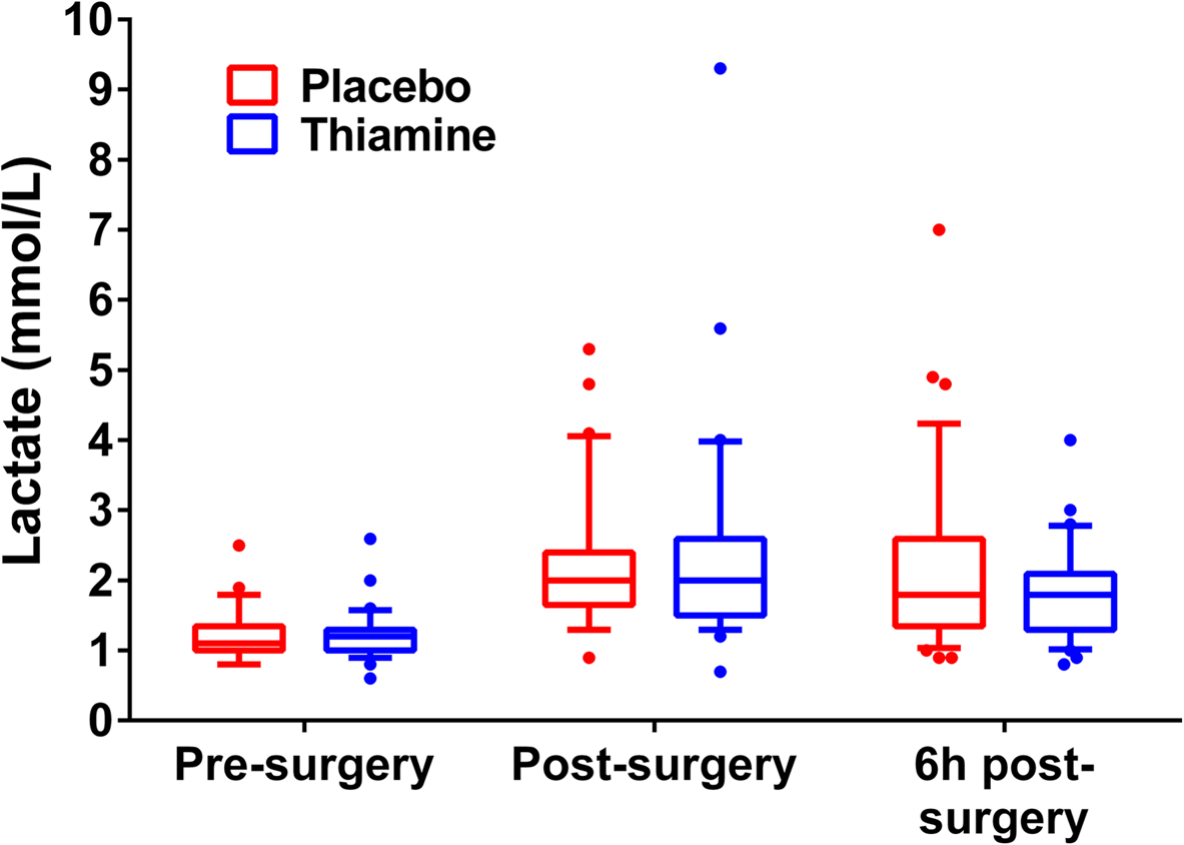
Lactate levels over time between the two groups. There was no difference between the thiamine and placebo groups in the primary endpoint of lactate levels immediately after the surgery (2.0 [1.5, 2.6] mmol/L vs. 2.0 [1.7, 2.4], *p* = 0.75). The boxplots represent the 1^st^ quartiles, median, and 3^rd^ quartile. The whiskers represent the 10^th^ and 90^th^ percentile and outliers are marked with dots

There was no difference between the thiamine and placebo groups in the primary endpoint of lactate levels immediately after the surgery (2.0 mmol/L [1.5, 2.6] vs. 2.0 mmol/L [1.7, 2.4], *p* = 0.75, Fig. 3). There was no difference in lactate levels 6 hours after the surgery (1.8 mmol/L [1.3, 2.1] vs. 1.8 mmol/L [1.4, 2.6], *p* = 0.45, Fig. 3). There was no difference in post-operative lactate levels when analyzed in the repeated measures model (*p* = 0.76).

**Fig. 4:**
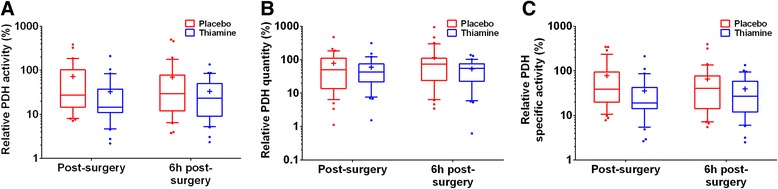
PDH values. Relative PDH activity (**a**), quantity (**b**) and specific activity (**c**) post-surgery and 6 hours post-surgery. Values were calculated as relative to the pre-surgery level, which was set at 100 %. The y-axis is logarithmic to better illustrate the findings. The boxplots represent the 1^st^ quartiles, median, and 3^rd^ quartile. The whiskers represent the 10^th^ and 90^th^ percentile and outliers are marked with dots

The PDH values are presented in Fig. 4. Relative PDH activity was significantly lower immediately after the surgery in the thiamine group as compared to the placebo group (15 % [11, 37] vs. 28 % [15, 84], *p* = 0.02). There was no difference 6 hours after the surgery (23 % [9, 50] vs. 29 % [12, 78], *p* = 0.19). There was no difference in relative PDH quantity immediately after the surgery (43 % [22, 75] vs. 50 % [15, 96], *p* = 0.93) or 6 hours after the surgery (56 % [23, 75] vs. 74 % [24, 111], *p* = 0.27). Relative PDH specific activity was lower in the thiamine group immediately after surgery (19 % [14, 42] vs. 39 % [20, 87], *p* = 0.01), but there was no difference 6 hours after surgery (27 % [12, 59] vs. 41 % [14, 77], *p* = 0.23). Given some imbalance between groups in PDH activity at baseline, we performed a post hoc analysis including the baseline PDH activity in the model. In this model, the difference between the two groups in relative PDH activity immediately after the surgery was not significant (*p* = 0.12). A post hoc comparison of absolute PDH values are presented in Additional file 1. There was no group difference between any of the absolute PDH values.

### Oxygen consumption

Global oxygen consumption (VO_2_) was measured on 27 patients: 15 in the placebo group and 12 in the thiamine group. There was no significant difference in baseline characteristics between those who had and those who did not have VO_2_ measured (see Additional file 1). There was a significant difference in VO_2_ 1 hour after surgery with the thiamine group having higher values (difference: 0.37 mL/min/kg [95 % CI: 0.03, 0.71], *p* = 0.03). There was no difference between the groups in change in VO_2_ over time from 1 to 4 hours after the surgery (*p* = 0.36). When the non-significant interaction was removed from the model, the between-group difference remained (difference: 0.19 mL/min/kg [95 % CI: 0.05, 0.33], *p* = 0.01) indicating that patients in the thiamine group had consistently higher VO_2_ values from one to four hours after the surgery.

**Fig. 5:**
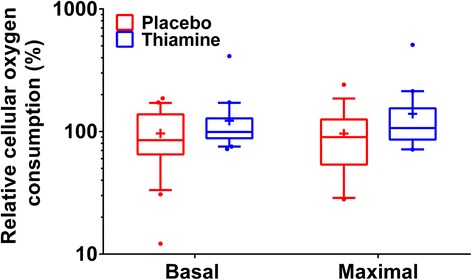
Cellular oxygen consumption. We found a significant difference in post-surgery relative basal oxygen consumption between groups (99 % [89, 126] vs. 85 % [66, 136], *p* = 0.04) and a significant difference in cellular maximal oxygen consumption between groups (107 % [86, 155] vs. 90 % [54, 125], *p* = 0.02). The boxplots represent the 1^st^ quartiles, median, and 3^rd^ quartile. The whiskers represent the 10^th^ and 90^th^ percentile and outliers are marked with dots. The y-axis is logarithmic to better illustrate the findings

Basal cellular oxygen consumption was available on 40 patients (20 in the placebo group and 20 in the thiamine group). There was no significant difference in baseline characteristics between those who had and those who did not have cellular oxygen consumption measured except for a slightly higher EuroSCORE II in those without cellular oxygen consumption measured (see Additional file 1). We found a significant difference in post-operative relative basal oxygen consumption between the thiamine and placebo group (99 % [89, 126], vs. 85 % [66, 136] *p* = 0.04, Fig. 5). Maximal cellular oxygen consumption was available on 38 patients (19 in the placebo group and 19 in the thiamine group). We found a significant difference in post-operative relative maximal oxygen consumption between the thiamine and placebo group (107 % [86, 155], vs. 90 % [54, 125], *p* = 0.02, Fig. 5).

### Clinical outcomes

There was no difference in time to extubation (8.3 hours [5.0, 19.2] vs. 7.4 hours [5.5, 12.3], *p* = 0.51) or time on vasopressors (12.8 hours [4.0, 21.1] vs. 9.3 hours [2.1, 22.1], *p* = 0.70) between the thiamine and placebo group. There was no difference in intensive care unit length of stay (2.4 days [1.1, 4.2] vs. 2.3 days [1.4, 4.0], *p* = 0.66) or in hospital length of stay (5 days [4, 8] vs. 5 days [4, 7], *p* = 0.49). There was no difference between the two groups in post-operative complications (see Table 3). One patient died in the placebo group and none in the thiamine group. No side effects of the study medication were reported in either group.

**Table 3:** Post-operative complications

	Thiamine (n = 31)	Placebo (n = 33)	*p* value
Atrial fibrillation	12 (39)	12 (36)	1.00
Renal failure	1 (3)	2 (6)	1.00
Stroke	0 (0)	0 (0)	1.00
Myocardial infarction	0 (0)	0 (0)	1.00
Acute respiratory distress syndrome	1 (3)	0 (0)	0.48
Infection	5 (16)	5 (15)	1.00
Delirium	3 (10)	4 (12)	1.00
At least one complication	16 (52)	16 (48)	1.00

### Subgroups analyses

Twenty-eight patients had a pre-surgery thiamine level below 14 mmol/L (the median of the entire cohort); 14 in each group. In this subgroup, there was no difference in the post-operative lactate level between those receiving thiamine and placebo (1.7 mmol/L [1.5, 2.4] vs. 2.1 mmol/L [1.6, 2.6], *p* = 0.32). Twenty-one patients had a EuroSCORE II above 4.0 %; 12 in the placebo group and nine in the thiamine group. There was no difference between the groups in post-operative lactate (3.0 mmol/L [2.1, 4.0] vs. 2.3 mmol/L [1.9, 3.5], *p* = 0.42) in this subgroup. Twenty-eight patients had diabetes; 14 in each group. There was no difference in post-operative lactate levels between groups (2.1 mmol/L [1.6, 3.3] vs. 2.0 mmol/L [1.7, 2.3], *p* = 0.43).

## Discussion

In this phase II trial, we found no difference in post-operative lactate levels or clinical outcomes between patients receiving thiamine or placebo. We did find a significant difference in post-operative cellular and global oxygen consumption between the two groups.

To our knowledge, this is the second randomized, placebo-controlled study to date to examine the efficacy of thiamine in this patient population. Recently, Luger et al. enrolled 30 patients undergoing cardiac surgery [47]. Patients were randomized to one dose of pre-operative thiamine (300 mg) or placebo. Similar to our findings, they found no difference in post-operative lactate levels or clinical outcomes. There are a few key differences between the study by Luger et al. and that presented here. First, we enrolled more than twice the number of patients. Second, we only included moderate- to high-risk patients (i.e., those with a EuroSCORE II > 1.5 %). Third, we provided two doses of thiamine: one before and one after the surgery. Lastly, we included a number of additional outcomes including PDH measurements, global and cellular oxygen consumption, and more granular clinical outcomes including an assessment of post-operative complications. Despite these differences, the findings were largely similar.

Donnino et al. recently found that thiamine did not decrease 24-hour lactate levels as compared to placebo in the overall group of patients (n = 88) with septic shock and elevated lactate (>3 mmol/L). However, in those with baseline thiamine deficiency (n = 28), thiamine improved 24-hour lactate levels as compared to placebo and there was a signal toward decreased mortality in the thiamine group [48]. In the current study, only one patient was thiamine deficient at baseline and it is possible that thiamine administration only has clinical utility in this subgroup of patients. Alternatively, thiamine could be beneficial for the group that develops thiamine deficiency during surgery but there is no way to assess this subgroup in the current study since provision of thiamine in the study arm precludes the capacity to determine who would have become deficient.

We used post-operative lactate levels as the primary outcome for multiple reasons. First, if thiamine were to improve PDH activity, as hypothesized, pyruvate should be converted to acetyl-coenzyme A leading to increased oxygen utilization and decreased lactate levels. Second, multiple studies have found an association between elevated post-operative lactate levels and increased morbidity and mortality [5–10]. In a recent study, we found that post-operative lactate levels were associated with hospital and intensive care unit length of stay as well as post-operative non-surgical complications. This association remained after adjusting for more than 25 patient and surgical characteristics indicating that lactate could be a suitable surrogate for more patient-centered outcomes [10]. Lastly, as a continuous outcome, lactate levels are a reasonable outcome in a phase II trial from a statistical point of view. However, the post-operative lactate levels in the placebo group were lower than anticipated minimizing the potential for a treatment effect.

The finding that relative PDH activity was decreased in the thiamine group was unanticipated, remains largely unexplained, and could be a chance finding potentially due to baseline imbalances (i.e., a post hoc analysis accounting for baseline PDH activity showed no significant difference between groups). One challenge in measuring PDH activity includes replicating the in vivo environment. The PDH assay we used, which has been described in detail elsewhere [38], measures PDH activity in vitro in an “ideal” environment which includes the addition of a small amount of thiamine in order for the reaction to run. The provision of in vitro thiamine (even at small levels) limits the between-group comparison and might therefore not reflect in vivo PDH values. Future studies examining the administration of thiamine or other modulators of PDH might consider using measures of in vivo PDH activity although these methods are considerably more complex and might not be feasible in the CABG population [49].

We found that thiamine significantly improved global oxygen consumption after CABG surgery. For an average 80 kg patient the increase would be approximately 30 mL/min. In an open-label trial of thiamine administration in the critically ill, Berg et al*.* found that thiamine improved oxygen consumption [50], consistent with the findings reported here. We also found that cellular oxygen consumption was increased in the group receiving thiamine. We are not aware of any previous studies examining the effect of thiamine (or other clinical interventions) on cellular oxygen consumption. Although the improvements in cellular and global oxygen consumption did not result in improved clinical outcomes in the current trial, future studies should examine whether improvements in oxygen consumption could improve patient-centered outcomes particularly in diseases where ongoing impairment of oxygen consumption is believed to be pathologic and causative of organ injury. The fact that we found a difference between groups in oxygen consumption, but no difference in lactate levels may indicate that lactate elevation in this context is not solely related to oxygen consumption but could reflect other mechanisms such as decreased clearance or excessive adrenergic stimulation [12, 51]. However, this remains speculative and will require additional studies.

The findings from the current study should be interpreted in the context of the study limitations. The sample size was relatively small and we might have been underpowered to detect differences between groups especially in the clinical outcomes. We only provided two doses of thiamine. While subsequent doses would not have affected our primary endpoint, they could affect more long-term outcomes. As noted above, the post-operative lactate levels were lower than expected and it is possible that including a more high-risk population with higher propensity for high post-operative lactate levels could have yielded different results. It is unknown whether measurements of PDH and oxygen consumption in PBMCs are representative of more relevant tissue such as the heart or the brain. Lastly, although the global oxygen consumption measurements used in this study has been validated [40, 41], this measurement is currently not considered the gold standard. The oxygen consumption results should be considered exploratory and hypothesis generating.

## Conclusions

In this randomized, placebo-controlled, double-blind, phase II trial, we found no difference in post-operative lactate levels or clinical outcomes between patients receiving thiamine or placebo. We found a significant increase in post-operative oxygen consumption in patients receiving thiamine.

## Key messages

- A randomized, double-blind, placebo-controlled trial of thiamine in 64 patients undergoing coronary artery bypass grafting was performed
- There was no difference in post-operative lactate levels between the two groups
- There was no difference in clinical outcomes between groups
- Patients in the thiamine groups had significantly higher post-operative cellular and global oxygen consumption

## Additional file

Additional file 1: Supplemental methods and results. (DOCX 477 kb)

## Abbreviations

CABG: Coronary artery bypass grafting
EuroSCORE: European System for Cardiac Operative Risk Evaluation
NYHA: New York Heart Association
OCR: Oxygen consumption rate
PBMC: Peripheral blood mononuclear cells
PDH: Pyruvate dehydrogenase
VO_2_: Global oxygen consumption
