# Supplementary material for: Thiamine as an adjunctive therapy in cardiac surgery: a randomized, double-blind, placebo-controlled, phase II trial
Source: Crit Care. 2016 Mar 14;20:92. doi: 10.1186/s13054-016-1245-1 (PMC4820988; doi:10.1186/s13054-016-1245-1)
Supplement: Additional file 1: — Supplemental methods and results. (DOCX 477 kb) [file 13054_2016_1245_MOESM1_ESM.docx]

**SUPPLEMENTAL METHODS**

*Measurement of cellular oxygen consumption*

In order to measure the cellular oxygen consumption rate (OCR) we utilized an XF^e^96 Extracellular Flux Analyzer (Seahorse Bioscience, North Billerica, MA, USA). Isolated peripheral blood mononuclear cells (PBMCs) were re-suspended in XF base medium supplemented with 5.5 mM glucose, 1mM sodium pyruvate and 4 mM L-glutamine. The PBMCs were then seeded in a 96-well assay plate (400,000 cells/well) pre-coated with cell adhesive (CellTak, BD Biosciences, San Jose, CA, USA) and centrifuged at 100 g for 3 min to allow for cells to attach to the bottom of the plate. The plate was then incubated at 37°C without CO_2_ for 30 minutes prior to assay start.

Modulators of respiration that target specific components of the electron transport chain (ETC) in the mitochondria were serially injected to reveal key parameters of metabolic function (XF Cell Stress Mito Kit, Seahorse Bioscience, North Billerica, MA, USA). First, OCR is measured for 3 cycles (see eFigure 1). Oligomycin, an inhibitor of ATP synthase (complex V), is then added. The decrease in OCR following injection of oligomycin correlates with the change in mitochondrial respiration associated with cellular ATP production. After another 3 measurement cycles, carbonyl cyanide-4 (trifluoromethoxy) phenylhydrazone (FCCP), an uncoupler of oxidative phosphorylation in the mitochondria, is added. FCCP collapses the proton gradient and disrupts the mitochondrial membrane potential. As a result, electron flow through the ETC is uninhibited and oxygen is maximally consumed by complex IV. The last injection, a mix of rotenone, a complex I inhibitor, and antimycin A, a complex Ill inhibitor, is added to inhibit electron transfer thereby completely inhibiting mitochondrial respiration.

3-5 replicates of each sample were measured, according to sample availability, and values were averaged across these samples. The various OCR parameters were defined as presented in eFigure 1 according with the manufacturer's recommendations. “Basal respiration” was calculated as the average of the three first measurements minus the non-mitochondrial respiration. “Non-mitochondrial respiration” was defined as the lowest OCR after injection of rotenone and Antimycin A. “Proton leak” was defined as the lowest OCR after injection of Oligomycin minus the non-mitochondrial respiration. “ATP production” was defined as the basal respiration minus the proton leak. “Maximal respiration” was defined as the highest OCR after injection of FCCP minus the non-mitochondrial respiration. Lastly, “spare capacity” was calculated as the difference between maximal and basal respiration. The protein concentration in each well was measured using a BCA protein assay (Thermo Scientific, Rockford, IL, USA) and the OCR values were subsequently normalized to the protein concentration before analysis. All values are expressed in pmol/min/μg protein. For this paper, we report the basal and maximal OCR.

Given that the cellular oxygen consumption measurements have to be done in real-time (i.e. samples cannot be stored/frozen for later analyses) and because the measurements require advanced equipment and trained personnel, measurements were generally not performed during nights and weekends where laboratory staffing was not available.


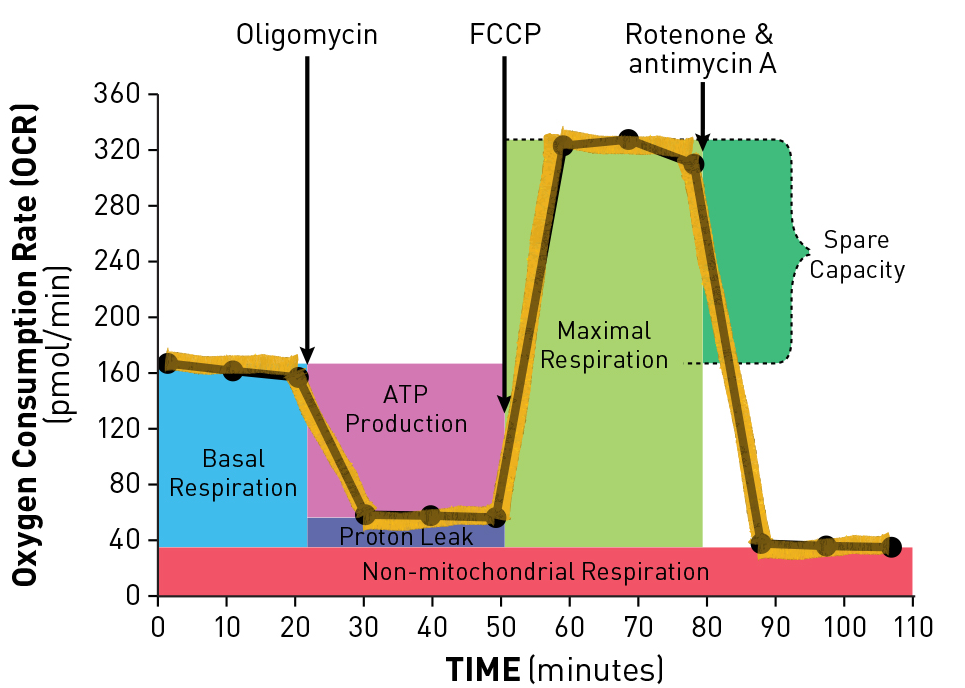


**eFigure 1. XF Cell Mito Stress Test Profile**

Schematic overview of cellular oxygen consumption using the XF Cell Mito Stress Test Profile kit in an XF^e^96 Extracellular Flux Analyzer (Seahorse Bioscience, North Billerica, MA, USA). Values in the current manuscript were normalized to the protein concentration and therefore do not correspond directly to those depicted on the y-axis. From www.seahorsebio.com with permission.

*Additional information regarding the sample size calculation*

The sample size calculation was based on data from a published observational trial[1] and an unpublished open-label, one-arm trial of thiamine administration. In the unpublished trial, all patients were administered 200 mg thiamine before their surgery. We applied the inclusion/exclusion criteria of the current trial to those two samples of patients. The non-thiamine group (n = 9) had post-operative lactate levels of 3.6 (standard deviation: 1.2 mmol/L) and the thiamine group (n = 8) had post-operative lactate levels of 2.6 (standard deviation: 0.6 mmol/L). To be conservative, we increased the estimated standard deviation to 1.4 mmol/L in both groups. With these estimates, for a two-sided t-test at an alpha of 0.05 and power of 80%, we estimated the need for 32 patients in each group in order to observe a statistically significant difference between groups.

**SUPPLEMENTAL RESULTS**

*Absolute PDH values*

 **eFigure 2. Absolute PDH values**

Absolute PDH activity (A), quantity (B) and specific activity (C) pre-surgery, post-surgery, and 6 hours post-surgery. The y-axis is logarithmic to better illustrate the findings. The boxplots represent the 1^st^ quartile, median, and 3^rd^ quartile. The whiskers represent the 10th and 90th percentile and outliers are marked with dots. The p-value is from the Wilcoxon Rank Sum test.

*Supplemental tables*

| **eTable 1. Baseline Characteristics of Patients with and without Global Oxygen Consumption Measurements^a,b^** | | | |
| --- | --- | --- | --- |
|  | **Patients without global oxygen consumption**  **(n = 37)** | **Patients with**  **global oxygen consumption**  **(n = 27)** | **p-value** |
| **Demographics** |  |  |  |
| Age (years) | 71 (67, 77) | 72 (65, 79) | 0.72 |
| Sex (female) | 12 (32) | 4 (15) | 0.15 |
| Body mass index (kg/m^2^) | 28 (25, 31) | 28 (26, 30) | 0.98 |
| Race |  |  | 1.00 |
| White | 36 (97) | 100 (100) |  |
| Black | 1 (3) | 0 (0) |  |
| **EuroSCORE II (%)** | 3.0 (1.9, 4.7) | 3.1 (2.1, 5.6) | 0.55 |
| **EuroSCORE II > 4.0 %** | 12 (32) | 9 (33) | 1.00 |
| **Cardiac past medical history** |  |  |  |
| MI/PCI | 13 (35) | 13 (48) | 0.32 |
| Atrial fibrillation | 9 (24) | 3 (11) | 0.21 |
| Previous cardiac surgery | 1 (3) | 1 (4) | 1.00 |
| Chronic heart failure | 5 (14) | 7 (26) | 0.33 |
| Valve disease | 10 (27) | 10 (37) | 0.42 |
| **Ejection fraction (%)** | 55 (45, 60) | 48 (33, 60) |  |
| **Current NYHA class^c^** |  |  | 0.45 |
| I | 4 (11) | 2 (7) |  |
| II | 13 (35) | 13 (48) |  |
| III | 17 (46) | 12 (44) |  |
| IV | 3 (8) | 0 (0) |  |
| **Current angina class^c^** |  |  | 0.82 |
| No symptoms | 16 (43) | 11 (41) |  |
| I | 0 (0) | 1 (4) |  |
| II | 5 (13) | 3 (11) |  |
| III | 9 (24) | 5 (19 ) |  |
| IV | 7 (19) | 7 (26) |  |
| **Other past medical history** |  |  |  |
| Pulmonary disease | 4 (11) | 4 (15) | 0.71 |
| Diabetes | 17 (46) | 11 (41) | 0.80 |
| Insulin dependent | 9 (24) | 4 (36) | 0.53 |
| Non-insulin dependent | 8 (22) | 7 (64) | 0.77 |
| Renal disease | 10 (27) | 8 (30) | 1.00 |
| Cancer | 0 (0) | 3 (11) | 0.07 |
| **Pre-operative laboratory values** |  |  |  |
| White blood count (x10^3^) | 7.9 (6.4, 8.8) | 7.9 (6.1, 10.2) | 0.60 |
| Hemoglobin (g/dL) | 12.9 (11.3, 14.5) | 12.9 (10.9, 14.2) | 0.56 |
| Creatinine (mg/dL) | 1.1 (0.8, 1.4) | 1.2 (0.9, 1.6) | 0.67 |
| Glucose (mg/dL) | 121 (100, 156) | 113 (90, 164) | 0.76 |
| **Pre-surgical characteristics** |  |  |  |
| Status |  |  | 1.00 |
| Elective | 15 (41) | 11 (41) |  |
| Urgent | 22 (59) | 16 (59) |  |
| Location prior to surgery |  |  | 1.00 |
| Home | 13 (35) | 9 (33) |  |
| Ward | 22 (59) | 17 (63) |  |
| Intensive care unit | 2 (5) | 1 (4) |  |

^a^ Categorical variables are presented as count (frequency) and continuous variables as median (quartiles).

^b^ EuroSCORE denotes European System for Cardiac Operative Risk Evaluation, MI myocardial infarction, PCI percutaneous coronary intervention and NYHA New York Heart Association.

^c^ Defined as the worst classification within the last two weeks.

| **eTable 2. Baseline Characteristics of Patients with and without Cellular Oxygen Consumption Measurements^a,b^** | | | |
| --- | --- | --- | --- |
|  | **Patients without cellular oxygen consumption**  **(n = 24)** | **Patients with**  **cellular oxygen consumption**  **(n = 40)** | **p-value** |
| **Demographics** |  |  |  |
| Age (years) | 75 (69, 81) | 71 (67, 77) | 0.11 |
| Sex (female) | 6 (25) | 10 (25) | 1.00 |
| Body mass index (kg/m^2^) | 26 (25, 29) | 29 (26, 33) | 0.05 |
| Race |  |  | 1.00 |
| White | 24 (100) | 39 (98) |  |
| Black | 0 (0) | 1 (3) |  |
| **EuroSCORE II (%)** | 3.9 (2.3, 5.7) | 2.5 (1.9, 4.0) | 0.03 |
| **EuroSCORE II > 4.0 %** | 11 (46) | 10 (25) | 0.10 |
| **Cardiac past medical history** |  |  |  |
| MI/PCI | 12 (50) | 14 (35) | 0.30 |
| Atrial fibrillation | 4 (17) | 8 (20) | 1.00 |
| Previous cardiac surgery | 1 (4) | 1 (3) | 1.00 |
| Chronic heart failure | 4 (17) | 8 (20) | 1.00 |
| Valve disease | 7 (29) | 13 (33) | 1.00 |
| **Ejection fraction (%)** | 55 (33, 60) | 55 (45, 60) | 0.37 |
| **Current NYHA class^c^** |  |  | 0.17 |
| I | 2 (8) | 4 (10) |  |
| II | 6 (25) | 20 (50) |  |
| III | 14 (58) | 15 (37) |  |
| IV | 2 (8) | 1 (3) |  |
| **Current angina class^c^** |  |  | 0.10 |
| No symptoms | 14 (58) | 13 (33) |  |
| I | 0 (0) | 1 (3) |  |
| II | 4 (17) | 4 (10) |  |
| III | 4 (17) | 10 (25) |  |
| IV | 2 (8) | 12 (30) |  |
| **Other past medical history** |  |  |  |
| Pulmonary disease | 3 (13) | 5 (13) | 1.00 |
| Diabetes | 7 (29) | 21 (53) | 0.08 |
| Insulin dependent | 3 (13) | 10 (25) | 0.34 |
| Non-insulin dependent | 4 (17( | 11 (28) | 0.38 |
| Renal disease | 6 (25) | 12 (30) | 0.78 |
| Cancer | 1 (40 | 2 (5) | 1.00 |
| **Pre-operative laboratory values** |  |  |  |
| White blood count (x10^3^) | 7.7 (6.6, 8.5) | 8.2 (6.0, 10.1) | 0.43 |
| Hemoglobin (g/dL) | 13.0 (11.6, 13.9) | 12.9 (11.2, 14.5) | 0.93 |
| Creatinine (mg/dL) | 1.1 (0.8, 1.1) | 1.2 (0.8, 1.6) | 0.331 |
| Glucose (mg/dL) | 106 (90, 141) | 135 (104, 172) | 0.06 |
| **Pre-surgical characteristics** |  |  |  |
| Status |  |  | 0.19 |
| Elective | 7 (29) | 19 (48) |  |
| Urgent | 17 (71) | 21 (53) |  |
| Location prior to surgery |  |  | 0.30 |
| Home | 6 (25) | 16 (40) |  |
| Ward | 16 (67) | 23 (58) |  |
| Intensive care unit | 2 (8) | 1 (3) |  |

^a^ Categorical variables are presented as count (frequency) and continuous variables as median (quartiles).

^b^ EuroSCORE denotes European System for Cardiac Operative Risk Evaluation, MI myocardial infarction, PCI percutaneous coronary intervention and NYHA New York Heart Association.

^c^ Defined as the worst classification within the last two weeks.

**References**

1. Andersen LW, Liu X, Peng TJ, Giberson TA, Khabbaz KR, Donnino MW: **Pyruvate Dehydrogenase Activity and Quantity Decreases After Coronary Artery Bypass Grafting: a Prospective Observational Study**. *Shock* 2015, **43**(3):250-254.
